# Synthesis of Carboxyl Modified Polyether Polysiloxane Surfactant for the Biodegradable Foam Fire Extinguishing Agents

**DOI:** 10.3390/molecules28083546

**Published:** 2023-04-18

**Authors:** Jinqing Jiao, Lei Qi, Jingfeng Wu, Xuqing Lang, Yuechang Wei, Guangwen Zhang, Pengyu Cui, Zuzheng Shang, Xiaodong Mu, Shanjun Mu, Yuzhuo Lv, Weichao Pan

**Affiliations:** 1 State Key Laboratory of Safety and Control for Chemicals, SINOPEC Research Institute of Safety Engineering Co., Ltd., Qingdao 266071, China; 2 State Key Laboratory of Heavy Oil Processing, China University of Petroleum (Beijing), Beijing 102249, China

**Keywords:** silicone surfactant, carboxyl modification, surface activity, structural performance, fluorine-free foam fire extinguishing agents

## Abstract

It is necessary to develop novel and efficient alternatives to fluorocarbon surfactant and prepare fluorine-free environmentally-friendly fire extinguishing agent. The carboxyl modified polyether polysiloxane surfactant (CMPS) with high surface activity was synthesized via the esterification reaction using hydroxyl-containing polyether modified polysiloxane (HPMS) and maleic anhydride (MA) as raw materials. The process conditions of the esterification reaction were optimized by orthogonal tests, and the optimum process parameters were determined as follows: reaction temperature of 85 °C, reaction time of 4.5 h, isopropyl alcohol content of 20% and the molar ratio of HPMS/MA of 1/1. The chemical structure, surface activity, aggregation behavior, foam properties, wetting properties and electron distribution were systematically investigated. It was found that the carboxyl group was successfully grafted into silicone molecule, and the conjugated system was formed, which changed the interaction force between the molecules and would affect the surface activity of the aqueous solution. The CMPS exhibited excellent surface activity and could effectively reduce the water’s surface tension to 18.46 mN/m. The CMPS formed spherical aggregates in aqueous solution, and the contact angle value of CMPS is 15.56°, illustrating that CMPS had excellent hydrophilicity and wetting performance. The CMPS can enhance the foam property and has good stability. The electron distribution results indicate that the introduced carboxyl groups are more inclined towards the negative charge band, which would be conducive to weak the interaction between molecules and improve the surface activity of the solution. Consequently, new foam fire extinguishing agents were prepared by using CMPS as a key component and they exhibited excellent fire-fighting performance. The prepared CMPS would be the optimal alternative to fluorocarbon surfactant and could be applied in foam extinguishing agents.

## 1. Introduction

The foam extinguishing agent is an important material for fire fighting [1,2,3]. Foam extinguishing agents can form a condensed foam floating layer on the surface of a flammable liquid and then successfully extinguish fire through its covering, cooling, isolation and suffocation effects. Fluorocarbon surfactants especially polyfluoroalkyl substances (PFAS), are the main surface-active materials in fire extinguishing agent [4,5,6]. However, recent studies have found that PFAS are difficult for the natural environment to decompose and are harmful to human bodies [7,8,9,10]. The study of PFAS decomposition is to reduce environmental hazards from the perspective of post-pollution treatment, while the search for PFAS substitutes is to reduce environmental hazards from the source; both of these are important, but the latter can permanently solve the environmental hazards. Therefore, it is necessary to develop novel alternatives to PFAS and efficient fluorine-free foam fire extinguishing agents [11,12,13,14,15].

Silicone surfactants were supposed to be the optimal alternatives to fluorocarbon surfactant because of their excellent surface activity, wettability, spreading properties, and biocompatibility [16,17,18,19,20,21,22,23,24]. Polyether modified silicone surfactant (PMS) is formed by the connection of the silicone chain segment and the polyether chain segment via chemical bond [25,26,27,28,29,30,31,32]. PMS is widely used in foam stabilizers, cosmetic materials, textile finishing agent and so on. However, it has low solubility and poor foaming ability, and is easy to break milk used in the foam extinguishing agent system. It is of great significance to carry out the study on the synthesis and properties of the novel silicone surfactants [33,34,35,36,37,38,39,40,41,42]. The chemical modification of silicones is an active research area, which could impart specific properties and trigger new applications by attaching several organic functional groups to the silicon atom [43,44,45,46,47,48]. Thus, many studies have been devoted to the modification of silicone surfactants, including amino modification, amino acid modification, carboxyl modification and so on [49,50,51]. Among various modification paths, the carboxyl modification has the most advantages in improving the surface properties of the silicone solution [52,53,54]. Until now, carboxyl modified polyether polysiloxane surfactants have been mostly side chain modification, and there are few reports on end group modification [55].

In the present paper, the carboxyl modified polyether polysiloxane surfactant (CMPS) were successfully prepared via esterification reaction using hydroxyl-containing polyether modified polysiloxane (HPMS) and maleic anhydride (MA) as raw materials. The process conditions were optimized by orthogonal test and the optimum process parameters were determined. In addtion, the chemical structure, surface activity, aggregation behavior, foam property and electron distribution were systematically investigated. New fluorine-free biodegradable foam fire extinguishing agents using CMPS as the key component were prepared and its fire extinguishing performance was measured.

## 2. Results and Discussions

### 2.1. Determination of the Optimum Experimental Condition

The optimum experimental condition for the synthesis of CMPS was determined by L16(4^3^) orthogonal test. Table 1 shows the orthogonal array experimental design and surface tension of the different CMPS aqueous solutions with different experimental conditions. Table 2 illustrates the analysis result from the perspective of surface tension, in which temperature, reaction time, solvent content and feeding ratio of the reactants are denoted as A, B, C and D.

As summarized in Table 2, it can be seen that the influence on surface tension decreases in the order of the reaction time > feeding ratio > solvent content > reaction time. Therefore, the optimum reaction condition schedule was determined as follows: reaction temperature of 85 °C, reaction time of 4.5 h, isopropyl alcohol (IPA) content of 20%, and the molar ratio of HPMS/MA of 1/1. Then the CMPS were synthesized under the optimal experimental conditions and structural characterization and surface properties were also performed.

### 2.2. FT-IR Spectra

**Figure 1:**
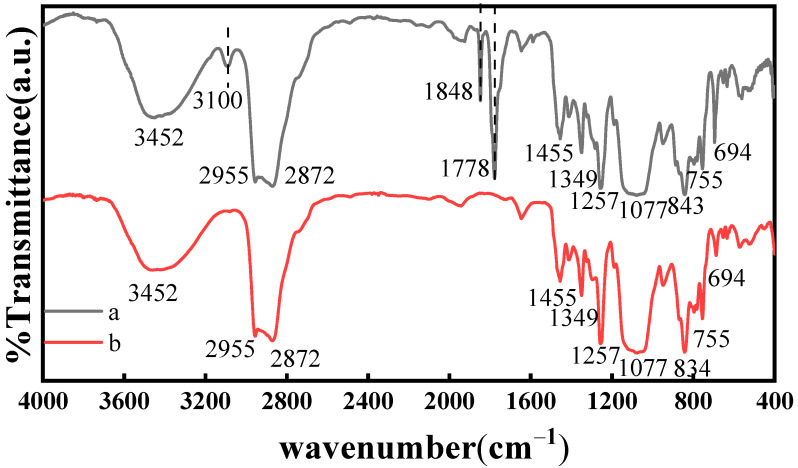
FT-IR spectra of CMPS (a) and HPMS (b) samples.

FT-IR spectra is an effective method with which to investigate the microstructure of samples. In FT-IR spectra, molecules with different functional group structures absorb infrared light at specific wavelengths, so the changes in the structures of molecular functional groups can cause changes in the location and intensity of the characteristic peaks. The FT-IR spectra of CMPS and HPMS are illustrated in Figure 1. As shown in Figure 1, CMPS and HPMS have similar peak positions and shapes. The absorption band at 3452 cm^−1^ is due to the stretching vibration of the -OH groups and the characteristic peak at 2955, 2872 and 1455 cm^−1^ are consistent with the stretching vibration of the -CH_2_, -CH_3_ groups. The absorption band at 1349, 1257 and 1077 cm^−1^ were ascribed to the stretching vibration of the -C-C-O- groups, -Si-C- groups and -C-O-C- groups, respectively. In addition, the difference between the CMPS and HPMS is that CMPS has sharper peaks at 1778, 1848 and 3100 cm^−1^. These three characteristic peaks are attributed to the -C=O- stretching vibration and the -C=C- stretching vibration, respectively. In conclusion, the FT-IR spectra indicated that the carboxyl group was successfully grafted onto the silicone molecule.

### 2.3. H-NMR Spectra

**Figure 2:**
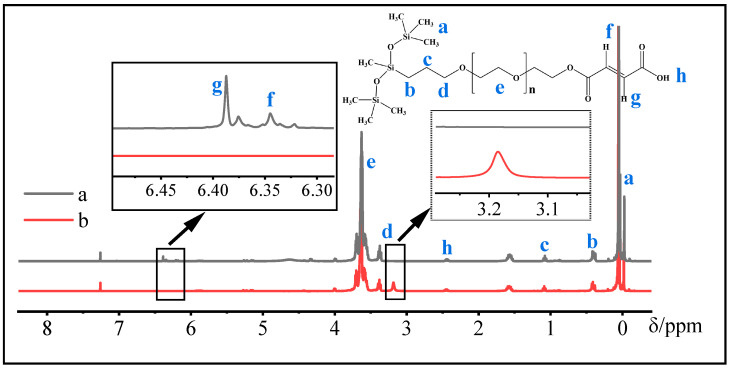
^1^H-NMR spectra of CMPS (a) and HPMS (b) samples.

The structure of two silicone surfactants was analyzed by ^1^H-NMR (400 MHz, CDCl_3_) and the results are shown in Figure 2. From Figure 2, there are three distinct peaks (at 6.40 ppm, 6.35 ppm, and 3.22 ppm) between two silicone surfactants. In detail, the peaks of the CMPS at 6.35 and 6.40 ppm are assigned to the proton of the -CH=CH- group. In addition, the peak at 3.22 ppm corresponds to the proton of the hydroxyl group (-OH) close to the methylene in the HPMS molecule. The above results suggest that the structure of CMPS is in agreement with the theoretical design, further proving that the carboxyl modified polyether polysiloxane surfactant has been successfully synthesized.

### 2.4. UV-Vis Absorption Spectra

**Figure 3:**
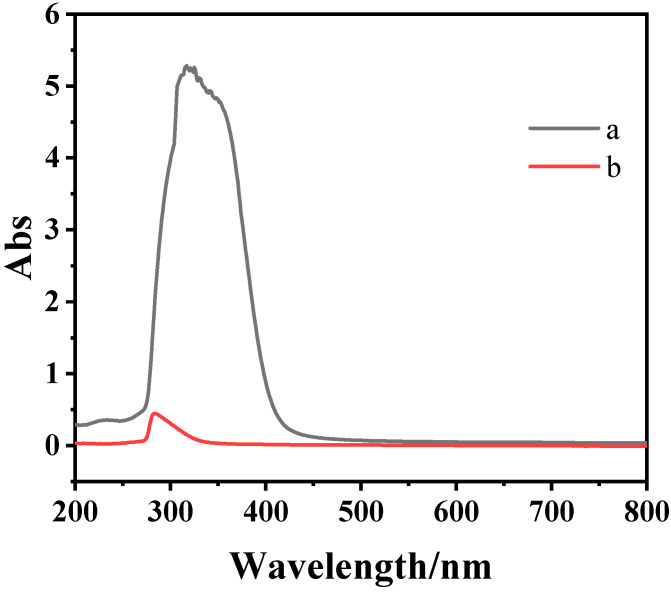
UV-Vis absorption spectra of CMPS (a) and HPMS (b) samples.

UV-Vis absorption spectra can accurately determine the molecular structure of organic compounds. The UV-Vis absorption spectra of the CMPS and HPMS samples are shown in Figure 3. As shown in Figure 3, it can be seen that CMPS exhibits stronger optical absorption in ultraviolet and visible regions as compared to that of HPMS. It is worthy of note that the CMPS has an obvious red-shift of the absorption edges. The enhancement of optical absorption intensity and red-shift for CMPS imply that the conjugated system is formed. The formed conjugated system may change the interaction force between the molecules and would affect surface activity of the silicone surfactant aqueous solution.

### 2.5. Surface Activity

**Figure 4:**
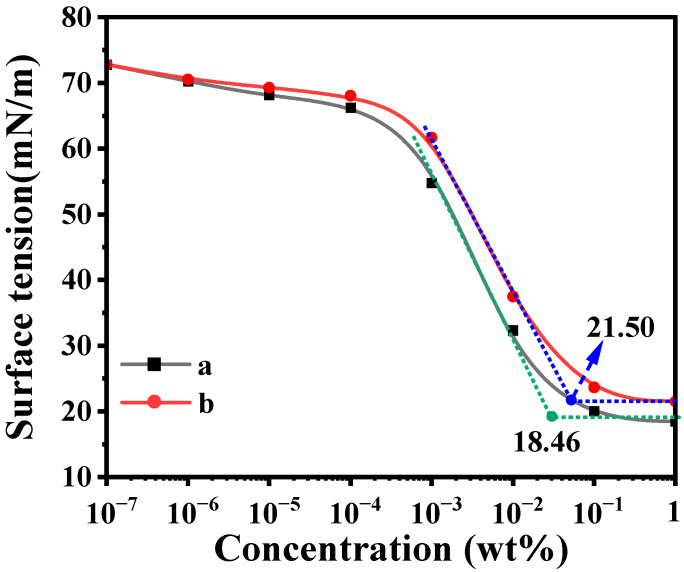
Surface tension of CMPS (a) and HPMS (b) aqueous solutions as a function of the mass fraction at 25 °C.

The surface activities in aqueous solution of CMPS and HPMS were evaluated by surface tension measurement. Figure 4 shows the relationship between the surface tension and mass fraction of two silicone surfactants. As illustrated in Figure 4, the surface tension of the two silicone surfactants solutions decreases with the increase in the concentration and then reaches a plateau. This is mainly because when the concentration of the surfactant aqueous solution is low, the surfactant molecules are dispersed and some molecules are oriented on the surface of the aqueous solution. At the moment, the oleophilic group faces outward and the hydrophilic group faces inward, resulting in a surface adsorption phenomenon, which can significantly reduce the surface tension of the aqueous solution. When the mass concentration of the surfactant further increases, the surface adsorption reaches saturation, resulting in the surfactant molecules being unable to continue to enrich on the surface. Meanwhile the hydrophobic chain oleophilic group will self-polymerize in the solution and begin to form micelles. When the surfactant concentration is further increased, the surfactant molecules will form more micelles inside the solution but will not cause the surface tension of the solution to decrease. At this time, the solution concentration is the critical micelle concentration (CMC). The CMC of the silicone surfactant is obtained by extending the straight lines on both sides of the turning point of the curve. The CMC of the HPMS and CMPS are 0.05 wt% and 0.03 wt%, respectively, and the corresponding surface tension is 21.50 and 18.46 mN/m, respectively. These results indicate that the CMPS exhibits superior surface activity. The surface tension of CMPS is very close to that of commercial fluorocarbon surfactants such as Capstone FS-50 (the surface tension is 16.99 mN/m at CMC of 0.0126%) [56]. 

The higher surface activity of CMPS may derive from the formed conjugated groups and the change of the surface excess concentration. The formed conjugated groups could cause the electrons to delocalize and decrease the energy of the system. Several surfactant molecules could self-assemble into a structure with a relatively low interaction force at the surface layer of the aqueous solution via electronic interaction of the formed conjugate system. That is to say, the interaction forces between the molecules were weakened, which would increase the number of aggregated molecules at the interface of the aqueous solution, and then improve the surface activity. In fact, the surface excess concentration (Γ_max_) and the area occupied by a single silicone surfactant molecule at the air/solution interface (A_min_) can be estimated by the Gibbs adsorption isotherm [57]. The detailed calculation process and results are listed in Figure S1 and Table S2. It is demonstrated that CMPS has a higher value of Γ_max_ (4.53 μmol/m^2^) and lower values of A_min_ (36.67 Å^2^) compared with that of HPMS, which indicated a denser arrangement of surfactant molecules at the air/solution interface.

### 2.6. TEM Results

**Figure 5:**
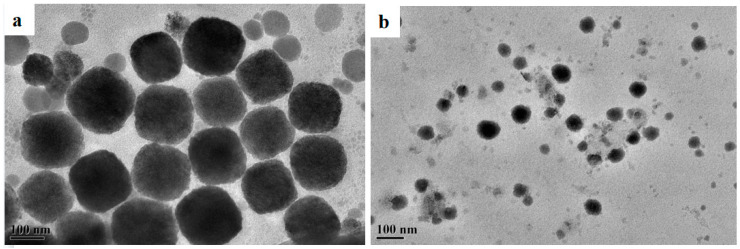
TEM images of aggregation of CMPS (**a**)and HPMS (**b**) samples.

In order to evaluate the aggregation states and morphologies of CMPS and HPMS in the aqueous solution, TEM characterizations were conducted and the results are shown in Figure 5. As illustrated in Figure 5, two silicone surfactants were self-assembled into spherical aggregates in aqueous solution. In addition, the size of spherical aggregates for CMPS ranged from 150 to 180 nm, and that for HPMS ranged from 20 to 80 nm. It can be clearly stated that different hydrophilic structures of silicone surfactants have a significant effect on their aggregates morphology. The formation of spherical aggregates was similar to the observation by Huang and Chen et al., which may be due to the hydrogen bonding or van der Waals interactions among the hydrophilic surfaces [57,58,59,60].

### 2.7. Wetting Properties

**Figure 6:**
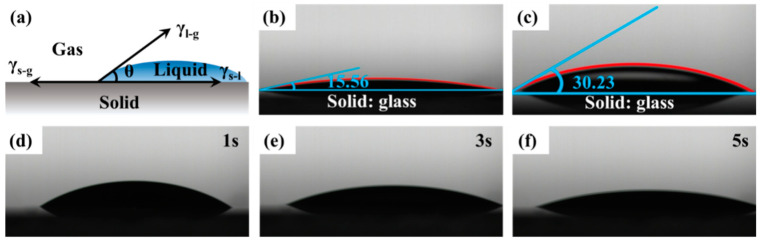
(**a**) Diagram of the contact angle of a droplet (γ_s−g_, γ_l−s_, and γ_l−g_ represented the interface tension of solid-gas, liquid-solid, and liquid-gas, respectively, and θ represents the contact angle); (**b**) The contact angle of the CMPS; (**c**) The contact angle of the HPMS; (**d**–**f**) The contact angle of the CMPS at different times.

The contact angle is the angle between the gas-liquid interface and the solid-liquid interface, as shown in Figure 6a. The contact angle of the CMPS on the glass surface was measured and the results are illustrated in Figure 6. As shown in Figure 6b,c, the contact angle of the silicone surfactant was reduced from 30.23° for HPMS to 15.56° for CMPS, indicating that CMPS has excellent wetting ability and superior hydrophilicity. The superior wetting ability and hydrophilicity are attributed to the low surface tension, the weak interaction force, the weak aggregation force and the low surface energy.

### 2.8. Foam Property

**Figure 7:**
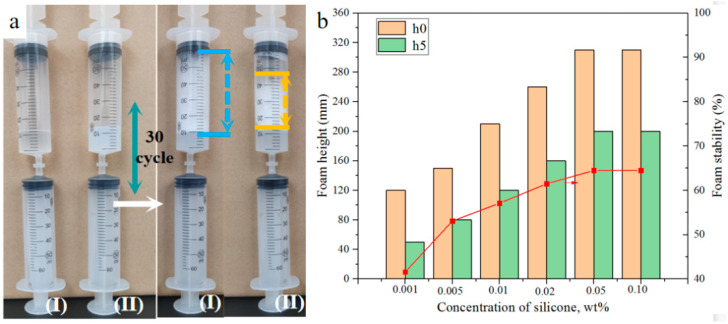
Foam property of CMPS (I) and HPMS (II) measured by the double-syringe technique (**a**) and the foam property of CMPS measured by Ross-Miles method (**b**).

The foam properties of CMPS and HPMS are measured by the double-syringe technique and the Ross-Miles method. Figure 7a illustrates the initial state and the foam after several pushes by the double-syringe technique. It can be seen that foam ability of the CMPS is higher compared with HPMS, mainly due to its lower surface tension. In addition, the CMPS aqueous solution is transparent and the HPMS aqueous solution is cloudy, which also indicates CMPS has superior hydrophilicity. Figure 7b and Figure S2 show the foam ability (h0, h5) and foam stability (R_5_) of CMPS and HPMS as a function of concentration. The foaming ability of CMPS and HPMS increase with the increase in the concentration, and then reaches a plateau. The foaming ability and foam stability of the CMPS aqueous solution reached the maximum value of 320 mL and 64.5% at the mass concentration of 0.05%, which is superior to that of HPMS. The above results indicate that CMPS effectively enhances the foam property and has good stability, which would be helpful for foam extinguishing agent with excellent foaming properties.

### 2.9. Electron Distribution Study

**Figure 8:**
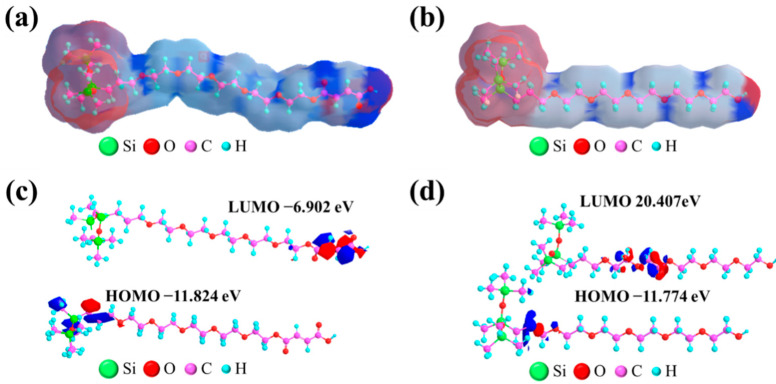
Schematic diagram of charge distribution (**a**,**b**) and energy levels (**c**,**d**) of CMPS (**a**,**c**) and HPMS (**b**,**d**).

Based on the above experimental results, the electron distribution of CMPS and HPMS were analyzed by Chem3D software using coulomb integral, exchange integral and overlap integral in combined with the Hueckel molecular orbit theory. The calculations results are shown in Figure 8 [61]. The red area represents the relatively positive area, and the blue area represents the relatively negative area. As illustrated in Figure 8a,b, the CMPS has more blue area on the end group position, indicating the structure is more inclined towards the negative charge band in comparison with HPMS. The above results are ascribed to the presence of a carboxyl group at the end of the hydrophilic chain, which has high electron adsorption capacity. 

In addition, the energy levels of the frontier molecular orbitals of CMPS and HPMS were studied as shown in Figure 8c,d. HOMO is the highest occupied molecular orbital, and LUMO is the lowest unoccupied molecular orbital. As illustrated in Figure 8c, the LUMO and HOMO levels of CMPS are −6.902 eV and −11.824 eV, respectively, and the energy level difference is 4.992 eV. The LUMO and HOMO levels of HPMS are 20.407 eV and −11.774 eV, respectively, and the energy level difference is 32.181 eV. From the perspective of electron distribution location, the HOMO orbitals of the two silicone surfactants are similar and are displayed in the hydrophobic siloxane chain segment. However, there are obvious difference in the point of the LUMO orbital. The LUMO distribution of HPMS is in the middle of the molecule, while the LUMO of CMPS is close to the carboxyl group, i.e., the conjugated group structure. It turns out that the more negative the LUMO orbital is, the easier it is for the LUMO orbital to accept electrons. The results are consistent with the above calculated electronic density distribution. The difference in electron distribution and system energy corresponds to the UV-Vis absorption spectra, which may affect surface activity of silicone surfactant aqueous solution.

### 2.10. Fire Extinguishing Performance and Biodegradability of Foam Fire Extinguishing Agent

**Figure 9:**
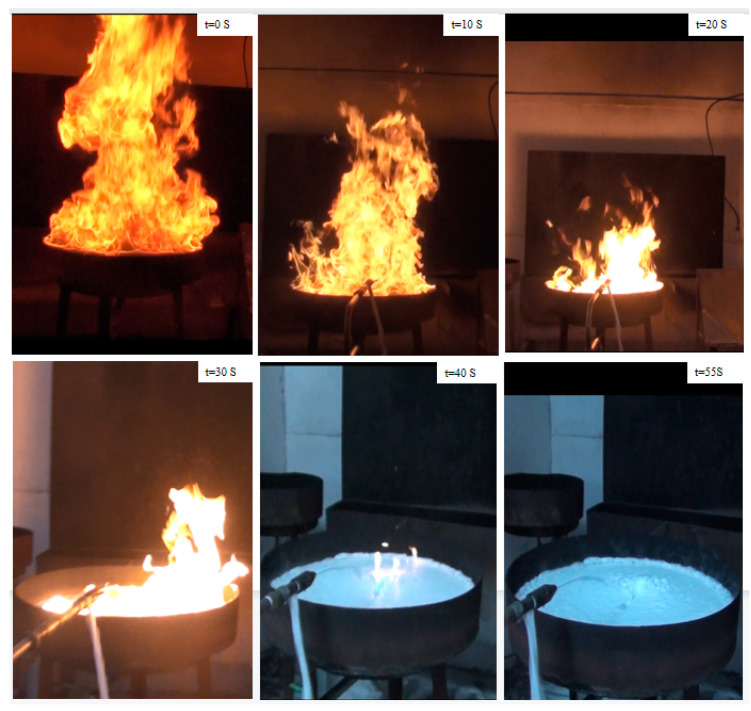
The interaction between flame and foams during fire extinguishing test of the foam-CMPS sample.

New foam fire extinguishing agents were prepared by using CMPS and HPMS as key components, which were denoted as foam-CMPS and foam-HPMS, respectively. The fire extinguishing effectiveness and fire retardancy of the environmentally-friendly fire extinguishing agent were measured according to the Chinese standard GB15308-2006 “Foam Fire Extinguishing Agent”, which is exactly the same as the standards BS EN 1568-3-2008. The scene picture of foam-CMPS and foam-HPMS were illustrated in Figure 9 and Figure S3, respectively. As shown in Figure 9, the flame height decreased obviously and the fire was gradually controlled after the foam extinguishing agent was injected into the fire tray for 20 s. Then the foam began to cover the oil surface and the flame height decreased obviously with increasing foams. With the foam further applied, the thickness of the foam layer on the fuel surface increases and the flame is gradually controlled and eventually extinguished. The fire extinguishing time of the foam-CMPS is 55 s. However, foam-HPMS failed to put out the fire after the foam applied for 180 s (Figure S3). The fire extinguishing performance indicate that foam-CMPS exhibits better fire-fighting property than the foam-HPMS. And the fire retardancy study of foam-CMPS was measured and the scene pictures were shown in Figure S4. The 25% burn-back time of the foam-CMPS is 15.6 min, which demonstrates the foam extinguishing agent has excellent fire resistance. The excellent fire extinguishing performance can be attributed to the key component CMPS, which has high surface activity, good foam property, superior foam stability and superior hydrophilicity. The biodegradability of foam fire extinguishing agent was measured according to the Chinese standard GB21801-2008 “Chemicals-Ready biodegradability-Manometric respirometry test”. According to the standard requirements, the value of biochemical oxygen demand (BOD) and chemical oxygen demand (COD) were measured. And the ratio of BOD and COD was calculated, which could represent the biodegradability. After testing, we found that the biodegradability of foam was as high as 86.0%, which was better than that of fluorine-containing foam extinguishing agents [62,63,64]. The good biodegradability of the foam extinguishing agent is mainly attributed to the large proportion of biodegradable components in the formulation and without polyfluoroalkyl substances.

## 3. Materials and Methods

### 3.1. Materials

**Figure 10:**
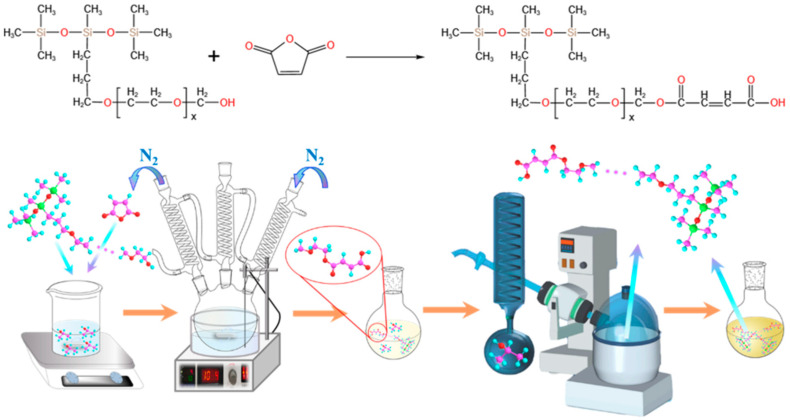
Reaction equation and the schematic for the synthesis of CMPS samples.

Hydroxyl-containing polyether modified polysiloxane (The detailed molecular structure is presented in Figure 10, where the “x” is about 8 and the average molecular weight is 663 g/mol) was purchased from Zhejiang Runhe Organic Silicon New Material Co., Ltd. (Huzhou, China). Maleic anhydride (MA) was purchased from Energy Chemical (Shanghai, China). Isopropyl alcohol (IPA) was obtained from Shanghai Macklin Biochemical Co., Ltd. (Shanghai, China). Deionized water was made in the laboratory.

The raw material for the preparation of the foam fire extinguishing agents are as follows: ethylene glycol, diethylene glycol butyl ether, urea, citric acid, sodium benzoate, fatty alcohol polyoxyethylene ether, sodium dodecyl sulfate and xanthan gum. They were purchased from Sinophurt Chemical Reagent Co., LTD (Shanghai, China). Alkyl glycosides and coactam betaine were purchased from Qingdao Huayuan Polymer Co., LTD (Qingdao, China).

### 3.2. Sample Synthesis

The CMPS was synthesized in a three-necked flask under a nitrogen atmosphere. The reaction equation and preparation process are illustrated in Figure 10. The typical synthesis processes were as follows: A certain quality of HPMS, maleic anhydride and isopropyl alcohol were mixed and stirred for 15 min at 25 °C. Then the mixture was transferred into a three-necked flask under the condition of magnetic stirring. The reaction temperature was adjusted in the range of 60–90 °C and the mixture was stirred for 2–6 h. Then the solvent was removed by vacuum distillation in a rotary evaporator. Finally, CMPS samples were obtained. Table 3 shows four important variables for the synthesis of CMPS, including temperature, reaction time, solvent content and feeding ratio of reactants. Determination of the optimal reaction conditions for CMPS was performed with a L16(4^3^) orthogonal test. After conducting the designed experiment, the surface activity of the corresponding reaction products was measured. 

New foam fire extinguishing agents using CMPS and HPMS as key components were prepared and denoted as foam-CMPS and foam-HPMS, respectively. The preparation process of foam fire extinguishing agents is as follows and the composition of the foam formulations are listed in Table S1. 3 g of urea, 10 g of sodium dodecyl sulfate, 2 g of alkyl glycosides, 4 g of ethylene glycol and 1 g of CMPS (or HPMS) surfactant were added into 79.8 g of deionized water. The above solution was stirred at room temperature for 20 min. Afterwards, 0.05 g of xanthan gum were added to the solution, and the mixture was further stirred for another 10 min. Then 0.1 g of citric acid was added in order to adjust the pH of the above mixture to 7.0~8.5. Finally, 0.05 g of sodium benzoate was added and the fluorine-free foam extinguishing agent were collected after stirring for 5 min.

### 3.3. Characterization

The ^1^H NMR spectra were obtained using a Bruker 400 MHz spectrometer in chloroform (CDCl_3_). FT-IR spectra were recorded by using a Bruker FT-IR spectrometer. The samples were dispersed in anhydrous KBr pellets before testing. UV-Vis absorption spectra were recorded by using a UV-2600i spectrophotometer with deionized water as the comparison sample. Surface tension measurements were carried out at 298.15 K using a model BZY-1 tensiometer. A Dataphysics DCAT21 (Germany) was used to investigate the wetting ability and hydrophilicity of the surfactants. The glass slides were immersed in hydrochloric acid for 24 h, washed with deionized water, and dried at room temperature. The contact angle (θ) was investigated by spreading a 5 μL drop of surfactant solution on the substrate. The θ values were recorded after the drop had been sitting on the substrate for 30–50 s. The morphologies of surfactant aggregations were examined with JEM-2100 TEM (JEOL, Japan) at 200 kV. The mass concentration of CMPS and HPMS solutions is 2 wt%. The samples were prepared by dropping the solution on a carbon-coated grid. Phosphotungstic acid solution (2 wt%) was used as stain solvent. The foam ability and foam stability of the surfactants were evaluated with a Ross-Miles instrument. Foam ability is a parameter characterized by the initial foam height (h0) and foam stability is calculated by the ratio of the five-minute foam height (h5) to the initial foam height (h0). In addtion, the foam ability is also measured by the double-syringe technique, which consists of two connected syringes. The volume of the syringe is 60 mL and the diameter of the tube is 3 mm. The initial volume of liquid is 15 mL and the concentration of 0.1%. The foam is generated by pushing both liquid and gas repeatedly through the connecting tube. The solution is passed through the tube 30 times and then the homogeneous foam is formed. The electron distribution and energy levels of frontier molecular orbital were analyzed by Chem3D software. The fire extinguishing effectiveness and fire retardancy of environmentally-friendly fire extinguishing agent were measured according to the Chinese standard GB15308-2006 “Foam Fire Extinguishing Agent”. In addition, the biodegradability of the foam fire extinguishing agent was measured according to the Chinese standard GB21801-2008 “Chemicals-Ready biodegradability-Manometric respirometry test”.

## 4. Conclusions

The carboxyl modified polyether polysiloxane surfactant (CMPS) was successfully synthesized via esterification reaction using hydroxyl-containing polyether modified polysiloxane (HPMS) and maleic anhydride (MA) as raw materials. The optimum process parameters were determined as follows: reaction temperature of 85 °C, reaction time of 4.5 h, isopropyl alcohol content of 20% and the molar ratio of HPMS/MA of 1/1. The end group carboxyl modification effectively improves the surface activity, solubility and foaming ability. The enhanced surface activity and superior hydrophilicity may originate from the maximum excess surface concentration, conjugated system, the extended carbon chain structure, the weakened interaction between molecules and the introduced hydrophilic group onto the molecules. The foam fire extinguishing agent using CMPS as key component exhibits excellent fire extinguishing performance and good biodegradability. The prepared CMPS would be the optimal alternatives to fluorocarbon surfactants, and we propose that it should be used in efficient environmentally friendly foam fire extinguishing agents on the large scale.

## Figures and Tables

**Table 1:** Orthogonal array experimental design and the measured surface tension.

Experiment Number	Temperature (°C)	Reaction Time (h)	IPA Content (wt%)	n(HPMS):n(MA)	Surface Tension (mN/m)
1	95	4.5	20%	1:2	19.92
2	75	2.5	40%	1:1	19.24
3	85	1.5	40%	1:3	19.40
4	85	3.5	0%	2:1	19.28
5	65	1.5	10%	1:3	20.08
6	95	2.5	0%	2:1	20.54
7	75	4.5	0%	1:2	20.74
8	75	1.5	20%	2:1	19.78
9	65	4.5	40%	2:1	19.87
10	95	3.5	40%	1:3	21.10
11	75	3.5	10%	1:3	20.63
12	65	2.5	0%	1:2	19.30
13	95	1.5	10%	1:1	20.44
14	65	3.5	20%	1:1	19.43
15	85	2.5	20%	1:2	19.24
16	85	4.5	10%	1:1	19.35

**Table 2:** Analysis result of experiment designed with orthogonal array table.

Levels		A	B	C	D
Surface tension (mN/m)	K1	19.67	19.93	19.97	19.87
	K2	20.10	19.61	20.13	19.62
	K3	19.34	20.11	19.62	19.83
	K4	20.50	19.97	19.90	20.30
	R	1.16	0.50	0.51	0.69
Primary and secondary analysis	A > D > C > B				
Best combination	A3B2C3D2				

**Table 3:** Orthogonal experimental conditions of carboxyl-modified silicone surfactants.

Levels (%)	Temperature (°C)	Reaction Time (h)	Solvent Content (wt%)	Feeding Ratio (a:b)
1	65	1.5	0	2:1
2	75	2.5	10	1:1
3	85	3.5	20	1:2
4	95	4.5	40	1:3

## Data Availability

Available data are presented in the manuscript.

## Supplementary Materials

The following supporting information can be downloaded at: https://www.mdpi.com/article/10.3390/molecules28083546/s1. The detailed description of raw material, preparation process of foam fire extinguishing agents and Figure S1: Surface tension of CMPS (a) and HPMS (b) aqueous solutions as a function of the mass concentration at 25 °C. Figure S2: The foam property of HPMS measured by Ross-Miles method. Figure S3: The interaction between flame and foams during fire extinguishing test of the foam-HPMS sample. Figure S4: The interaction between flame and foams during burn-back test of the foam-CMPS sample. Table S1: Composition of the studied foam formulations. Table S2: The surface activity and adsorption parameters of CMPS and HPMS solutions.

## Sample Availability

Not available.
