# Supplementary material for: Synthesis of Carboxyl Modified Polyether Polysiloxane Surfactant for the Biodegradable Foam Fire Extinguishing Agents
Source: Molecules. 2023 Apr 18;28(8):3546. doi: 10.3390/molecules28083546 (PMC10142534; doi:10.3390/molecules28083546)
Supplement: Supplementary file 1 [file molecules-28-03546-s001.zip › molecules-2222250-supplementary.pdf]

Synthesis of Carboxyl Modified Polyether Polysiloxane Surfactant for the Biodegradable Foam Fire Extinguishing Agents

Jinqing Jiao [1,*], Lei Qi [2], Jingfeng Wu [1], Xuqing Lang [1], Yuechang Wei [2,*], Guangwen Zhang [1], Pengyu Cui [1], Zuzheng Shang [1], Xiaodong Mu [1], Shanjun Mu [1], Yuzhuo Lv [2] and Weichao Pan [2]

[1] State Key Laboratory of Safety and Control for Chemicals, SINOPEC Research Institute of Safety Engineering Co., Ltd., Qingdao 266071, China; wujf.qday@sinopec.com (J.W.); langxq.qday@sinopec.com (X.L.); zhanggw.qday@sinopec.com (G.Z.); cuipy.qday@sinopec.com (P.C.); shangzz.qday@sinopec.com (Z.S.); muxd.qday@sinopec.com (X.M.); mushanjun@VIP.Sina.com (S.M.)

[2] State Key Laboratory of Heavy Oil Processing, China University of Petroleum (Beijing), Beijing 102249, China; qileilw@163.com (L.Q.); lvyuzhuolw@163.com (Y.L.); marqpan98@gmail.com (W.P.)

* Correspondence: jiaojq.qday@sinopec.com (J.J.); weiyc@cup.edu.cn (Y.W.)

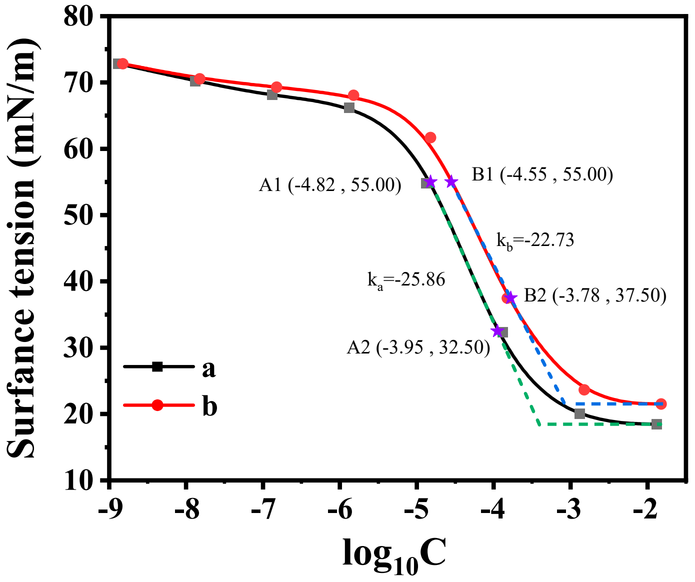

Figure S1. Surface tension of CMPS (a) and HPMS (b) aqueous solutions as a function of the mass concentration at 25 °C.

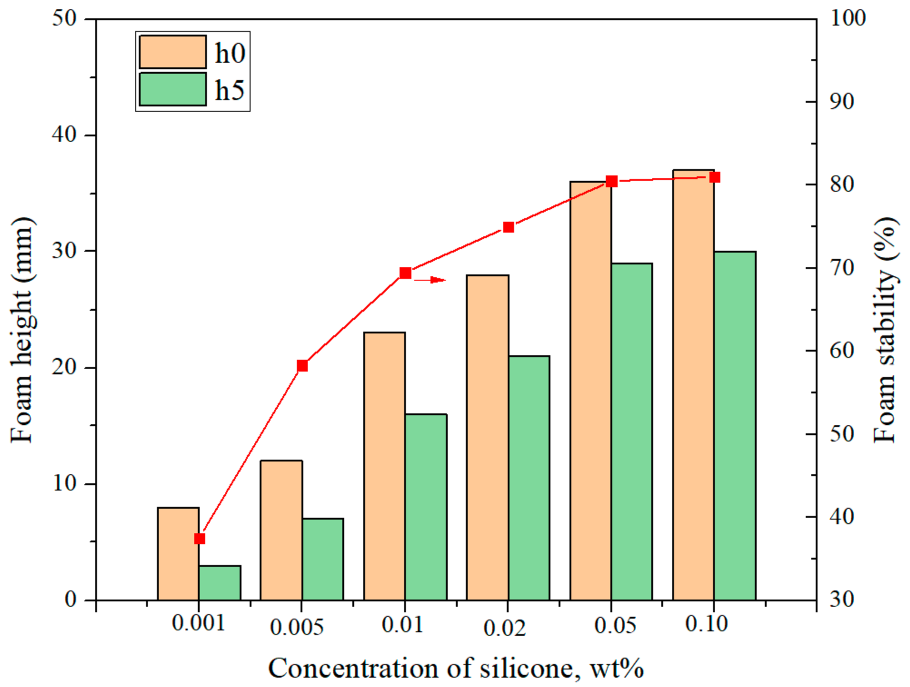

Figure S2. The foam property of HPMS measured by Ross-Miles method.

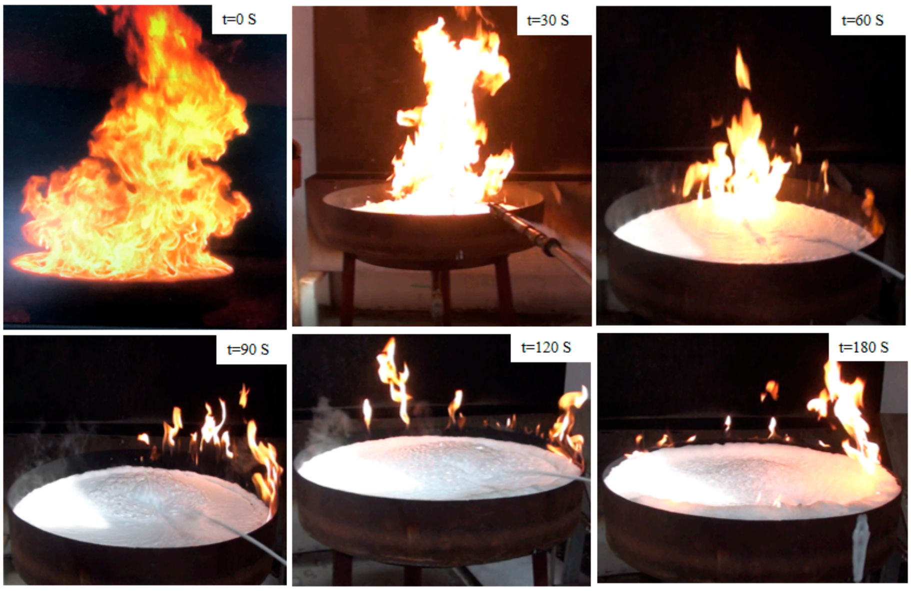

Figure S3. The interaction between flame and foams during fire extinguishing test of the foam-HPMS sample

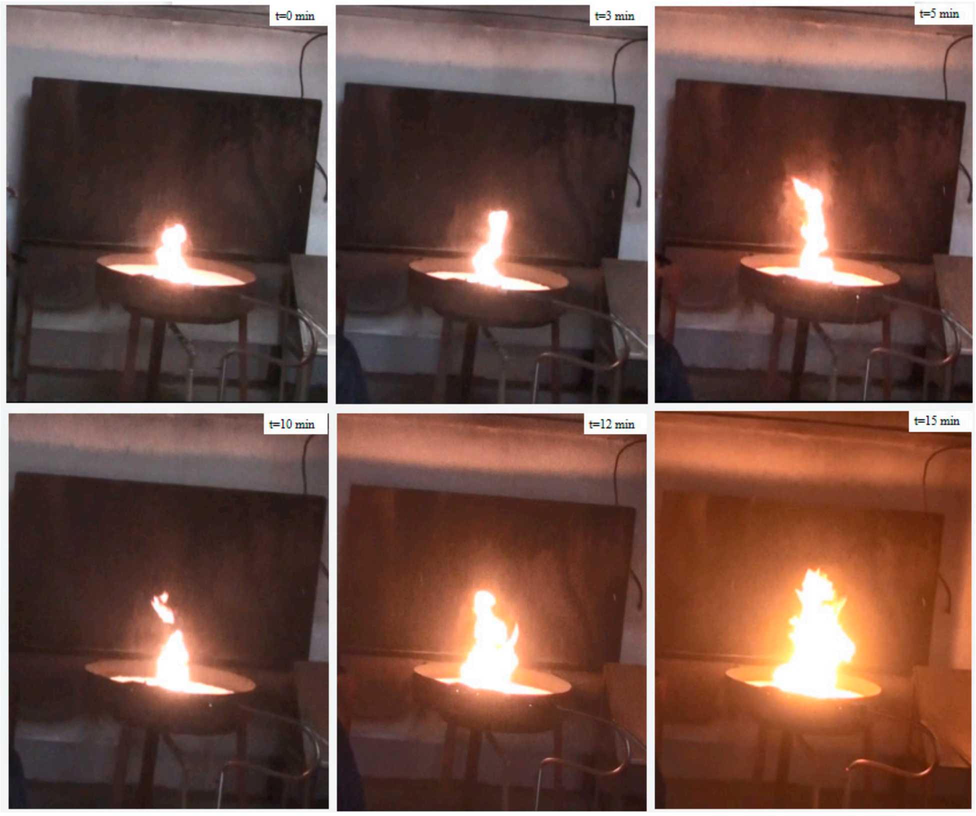

Figure S4. The interaction between flame and foams during burn-back test of the foam-CMPS sample.

Table S1 Composition of the studied foam formulations

**Material**	**Content (%)**
sodium dodecyl sulfate	10
urea	3
alkyl glycosides	2
CMPS (HPMS)	1
ethylene glycol	4
xanthan gum	0.05
citric acid	0.10
sodium benzoatethe	0.05
deionized water	79.8

Table S2. The surface activity and adsorption parameters of CMPS and HPMS solutions

Sample	γ_{cmc}(mN/m)	Γ_{max}(μmol/m^2)	A_{min}(Å^2)
CMPS	18.46	4.53	36.67
HPMS	21.50	3.98	41.74

The specific calculation process is as follows:

Firstly, we converted the mass fraction (**ω**) of silicone surfactant solution to the molar concentration (C) based on the data of Figure 5 and following equation (1-2). The relationship between the surface tension and molar concentration of two silicone surfactant are shown in Figure S1.

$$C = \frac{n}{\mathrm{V}} \qquad n = \frac{m}{\mathrm{M}} \qquad m = \rho \cdot \mathrm{v} \cdot \boldsymbol{\omega} \qquad (1)$$

$$C = \frac{1000\rho\boldsymbol{\omega}}{\mathrm{M}} \qquad (2)$$

Where C is the molar concentration (mol/L), n is amount of substance (mol), m is mass of substance (g), V is the solution volume (cm^3), **ω** is mass fraction(%), M is relative atomic mass of silicone surfactant and the value of HPMS and CMPS are about 663 and 761 g/mol, ϱ is the density of silicone solution, the value is approximately equal to the density of water (1g/cm^3).

As illustrated in Figure S1, the surface tension gradually decreases with increasing the silicone surfactant concentration and then reaches a plateau. That is to say that there is a breaking point in the γ-$\log_{10}C$ plot. The concentration of the break point was considered to be the critical micelle concentrations (CMC). The CMC of silicone surfactant is obtained by extending the straight lines on both sides of the breaking point of the curve.

The surface excess concentration (Γ_{max}) and the area occupied by a single silicone surfactant molecule at the air/solution interface (A_{min}) can be estimated by the Gibbs adsorption isotherm (equation 3-4).

$$\Gamma_{max} = -\frac{1}{2.303RT}\left(\frac{d\gamma}{dlog_{10}C}\right) \quad (3)$$

$$A_{min} = \frac{10^{16}}{N_A \cdot \Gamma_{max}} \quad (4)$$

where γ is the surface tension (mN/m, dyn/cm, ergs/cm^2), C is the concentration of surfactant (mol/L), R is the ideal gas constant (8.314×10^7 ergs/mol·K), T is the Kelvin temperature (K), $\frac{d\gamma}{dlog_{10}C}$ is the slope of the linear of the data before the CMC in the surface tension plots (ergs/cm^2) and N_A is Avogadro's constant (6.02×10^{23}/mol). The $\frac{d\gamma}{dlog_{10}C}$ value of CMPS and HPMS could be represented by the slope of green dotted line (k_a) and blue dotted line (k_b), respectively. For CMPS, take any two points on the slope line, such as A1(-4.82, 55.00), A2(-3.95, 32.50), and the k_a value can be calculated from the following equation.

$$\frac{d\gamma}{dlog_{10}C} = k_a = \frac{y_{A2}-y_{A1}}{x_{A2}-x_{A1}} = \frac{32.50-55.00}{-3.95-(-4.82)} = -25.86\ ergs/cm^2 \quad (5)$$

The k_b value of HPMS was calculated by the same method and the value was -22.73 ergs/cm^2. Thus, substituting the above values into the equation 3 and equation 4, and the Γ_{max} and A_{min} of CMPS and HPMS were calculated. The detailed calculation process for CMPS is as follows:

$$\Gamma_{max} = -\frac{1}{2.303 \times 8.314 \times 10^7 ergs/mol \cdot K \times 298.15K} \times (-25.86 ergs/cm^2)$$

$$= 4.53 \times 10^{-10} \text{mol/cm}^2 = 4.53\ \mu\text{mol/m}^2$$

$$A_{min} = \frac{10^{16}}{6.02 \times 10^{23}/mol \times 4.53 \times 10^{-10} mol \cdot cm^{-2}} = 36.67\text{Å}^2$$

The Γ_{max} and A_{min} of HPMS were calculated by the same method and the Γ_{max} was 3.98×10^{-10} mol/cm^2 (3.98 μmol/m^2) and the A_{min} is 41.74 Å^2.
